# Apocynum Cannabinum

**Published:** 1891-06

**Authors:** W. T. Richmond


For Daniel's Texas Medical Journal.

### APOCYNUM CANNABINUM.

BY W. T. RICHMOND, M. D.

[Read at the Austin District Medical Society.]

MY EXCUSE for calling your attention to apocynum cannabinum, is that the text-books have so little to say of an agent that I have found valuable. While some of them attribute medical properties to it, none that I have seen give it due credit in the treatment of dropsies.

For its origin, description and history, I must refer you to the dispensatories. I intend to speak of its use in the treatment of dropsies only.

The preparation I have used is the fluid extract. Thirty or forty drops will act promptly, as an emetic. I usually begin with seven or eight drops, and increase the dose, or repeat at short intervals, until the desired effect is obtained. This quantity will act on the bowels, producing copious watery discharges, and increase the flow of urine. Its action on the kidneys must be by increase of blood pressure as the action of the heart is slowed.

I believe it increases the flow of bile, as some cases of jaundice treated with it have yielded promptly, the stools becoming dark with the use of the medicine.

A case of acute nephritis with ascites, in a child ten years of age, was given three drops fluid extract apocynum every four hours until its bowels moved freely. Copious fluid discharges followed the third dose. On the following day, the urine contained less albumen, and the dropsy was less. The medicine was then given, in the same dose, three times a day, and in a week both the dropsy and albumen had entirely disappeared, and the child was much improved in every respect.

A case of chronic Bright's disease, without dropsy, was given seven drops of fluid extract apocynum three times a day, with instructions to increase the dose two drops every day until copious discharges from the bowels were produced.

When this patient began the use of the apocynum, her urine was loaded with albumen, her appearance cadaveric, nervous system impaired, and vision so defective that she could not recognize an acquaintance across the room. With the use of the apocynum, her general health improved rapidly, and in a few weeks the albumen had almost entirely disappeared from the urine. She became pregnant about this time, and the albumen reappeared in large quantities.

A patient having heart disease, with a loud systolic murmur, was treated with apocynum. About three months before commencing this treatment, it was necessary to tap her, to relieve the dispnœa caused by ascites and general anasarca. About four gallons of water was withdrawn. This relieved the dispnœa. The fluid reaccumulated rapidly, and second paracentesis had to be resorted to in less than three months, although compound jalap powder and other cathartics had been used. After the second tapping the fluid returned rapidly, and the patient became jaundiced. I then commenced giving fluid extract apocynum in ten drop doses, three times daily, with instructions to increase the dose until copious actions from the bowels were obtained. In a short time the jaundice disappeared, and in a month the dropsy was gone.

This patient is unable to take the apocynum constantly, on account of the nausea produced by it, and the dropsy returns when the medicine is discontinued.

From observation, I find that apocynum in small doses acts as a cathartic, without producing nausea or other disagreeable symptoms, but acts rather as a tonic and appetizer. It also acts as a diuretic. In large doses, or doses repeated at short intervals, it acts as a hydrogogue cathartic and diuretic. In large doses it is emetic. Its greatest value is in the treatment of dropsies from any cause, (especially from albuminuria).

Toleration is established by continued use, therefore it is necessary to increase the quantity given in order to maintain its action.

